# A Potent and Effective Suicidal *Listeria* Vaccine Platform

**DOI:** 10.1128/IAI.00144-19

**Published:** 2019-07-23

**Authors:** William G. Hanson, Erin L. Benanti, Edward E. Lemmens, Weiqun Liu, Justin Skoble, Meredith L. Leong, Chris S. Rae, Marcella Fassò, Dirk G. Brockstedt, Chen Chen, Daniel A. Portnoy, Thomas W. Dubensky, Peter Lauer

**Affiliations:** a Aduro Biotech, Inc., Berkeley, California, USA; b Department of Molecular and Cell Biology, University of California, Berkeley, Berkeley, California, USA; c Department of Plant and Microbial Biology, University of California, Berkeley, Berkeley, California, USA; University of Illinois at Chicago

**Keywords:** *Listeria monocytogenes*, biotechnology, cell-mediated immunity, immunotherapy, vaccines

## Abstract

Live-attenuated Listeria monocytogenes has shown encouraging potential as an immunotherapy platform in preclinical and clinical settings. However, additional safety measures will enable application across malignant and infectious diseases. Here, we describe a new vaccine platform, termed Lm-RIID (L. monocytogenes recombinase-induced intracellular death), that induces the deletion of genes required for bacterial viability yet maintains potent T cell responses to encoded antigens. Lm-RIID grows normally in broth but commits suicide inside host cells by inducing Cre recombinase and deleting essential genes flanked by *loxP* sites, resulting in a self-limiting infection even in immunocompromised mice. Lm-RIID vaccination of mice induces potent CD8^+^ T cells and protects against virulent challenges, similar to live L. monocytogenes vaccines. When combined with α-PD-1, Lm-RIID is as effective as live-attenuated L. monocytogenes in a therapeutic tumor model. This impressive efficacy, together with the increased clearance rate, makes Lm-RIID ideal for prophylactic immunization against diseases that require T cells for protection.

## Abstract (precis)

Live-attenuated Listeria monocytogenes has shown encouraging potential as an immunotherapy platform in preclinical and clinical settings. However, additional safety measures will enable application across malignant and infectious diseases. Here, we describe a new vaccine platform, termed Lm-RIID (L. monocytogenes recombinase-induced intracellular death), that induces the deletion of genes required for bacterial viability yet maintains potent T cell responses to encoded antigens.

## INTRODUCTION

Listeria monocytogenes is a Gram-positive intracellular pathogen that induces innate and adaptive immune responses in mice, enabling long-lived protection against itself (1) or viral (2, 3) or parasitic (4) challenge and therapeutic benefit in mouse tumor models (3, 5–10). Protection depends on CD8^+^ T cells (1, 2, 8, 11, 12), and, together with other advantages, such as straightforward manufacturing and the ability to repeatedly administer L. monocytogenes (7), has paved the way for clinical trials in advanced cancers (13–15). Key to the clinical advancement of L. monocytogenes vaccines has been the development of genetically defined live-attenuated vaccines that retain the immunologic potency of wild-type (WT) strains. However, with safety a paramount requirement for broad applicability, we have sought additional approaches to prevent pathogenesis while retaining the capacity to prime potent T cell immunity.

L. monocytogenes vaccine attenuation has been achieved through different strategies. A common approach involves virulence or metabolic gene deletion. Deletion of *actA*, which encodes a protein that mediates cell-to-cell spread, results in a strain that is 1,000-fold less virulent than WT L. monocytogenes in mice yet is capable of inducing protective immunity (5, 11). L. monocytogenes can also be attenuated through deletion of the gene encoding the virulence regulator PrfA, which is partially complemented when expressed from a plasmid. Both approaches have yielded vaccines with acceptable safety profiles in patients with advanced cancer (13, 14, 16).

An alternative attenuation strategy is engineering vaccines that cannot replicate in host cells. One example is an L. monocytogenes vaccine that undergoes lysis in host cells to facilitate DNA, RNA, and/or protein delivery (17–20). These strains rely on a bacteriophage lysin that is expressed only when L. monocytogenes reaches the host cytosol. Another example is KBMA (for killed but metabolically active) vaccines that consist of bacteria rendered sensitive to long-wavelength UV light by deletion of *uvrAB*, genes required for nucleotide excision repair (3). Low-level photochemical cross-linking of bacterial DNA renders KBMA bacteria unable to replicate, yet they access host cytosol and prime CD8^+^ T cells, leading to therapeutic benefits in a mouse tumor model. L. monocytogenes also has been attenuated by the deletion of genes required for synthesis of the cell wall component d-alanine (21). These strains require d-Ala for growth *in vitro* and to generate functional immune responses in mice yet do not replicate inside host cells. All three of these attenuation strategies are less potent than live L. monocytogenes (3, 6, 20–22), which is consistent with lysis in host cells being detrimental to immunogenicity (23).

Live-attenuated double-deleted L. monocytogenes (LADD) vaccines are being evaluated for the treatment of advanced-stage cancer. These vaccines are deleted for *actA* and *inlB* (5), a membrane-localized internalin required for hepatocyte invasion (24), and are cleared from mice by 4 days postimmunization yet induce robust immune responses similar to those of WT L. monocytogenes. Although LADD vaccines have been safely administered to more than 450 late-stage cancer patients (Aduro Biotech, Inc., unpublished data), further enhancing LADD vaccine safety could allow for its use in an even broader population (e.g., early-stage cancers and chronic infectious diseases).

The requirement for L. monocytogenes to be alive (replication competent) to induce potent immune responses presents a challenge in balancing attenuation while maintaining sufficient viability for vaccine potency. LADD vaccines induce immune responses due to their ability to enter and grow in the host cell cytosol, activate innate immunity, and express exogenous antigens that are processed and presented by host cells. However, it is unknown how long viability is required after antigen expression, and a loss in viability at later times postimmunization may increase vaccine safety. Here, we describe a new genetic strategy that leads to L. monocytogenes suicide in host cells. These strains, termed Lm-RIID for Listeria monocytogenes recombinase-induced intracellular death, excise essential genes upon entering the host cell cytosol. In mice, Lm-RIID strains are cleared faster than LADD strains from the liver and spleen yet induce similar immune responses, provide protective immunity against homologous (virulent) L. monocytogenes or heterologous vaccinia challenge, and confer therapeutic efficacy in a mouse tumor model. Combining Lm-RIID with α-PD-1 therapy increased survival to 100%, the same efficacy as that of LADD. The enhanced safety properties and robust potency make Lm-RIID an attractive alternative to existing vaccines.

## RESULTS

### Lm-RIID construction and growth.

**FIG 1:**
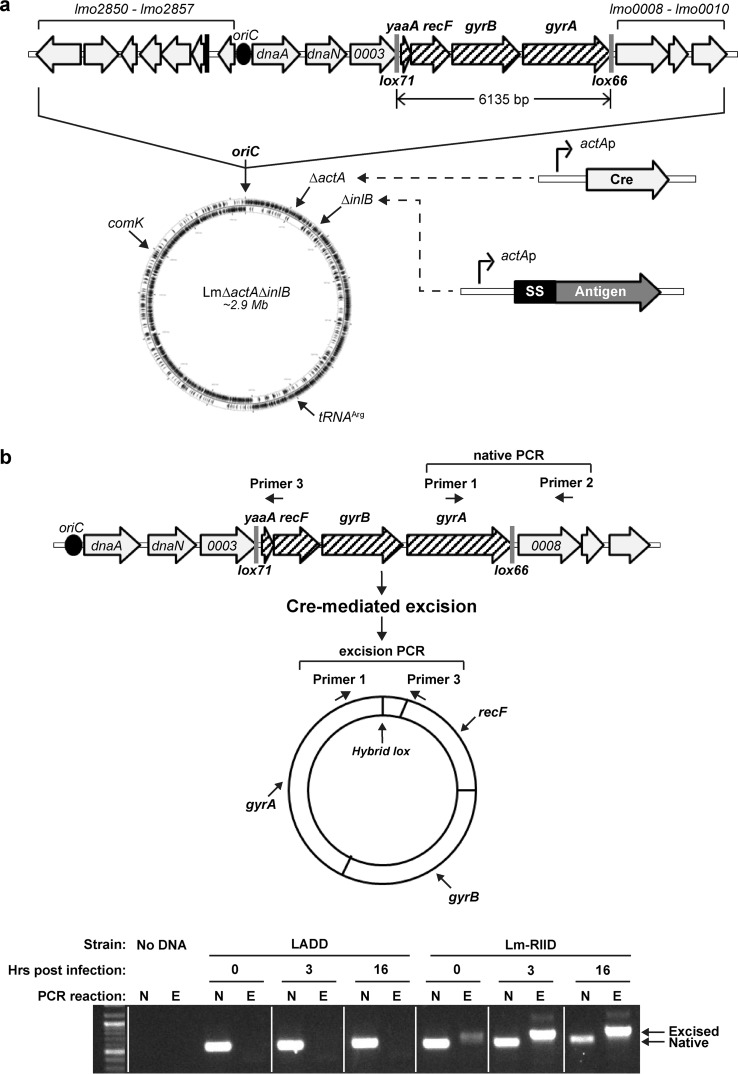
Suicidal L. monocytogenes (Lm-RIID) undergoes intracellular chromosomal excision. (a) Schematic overview of genomic alterations and cassettes in one L. monocytogenes suicide vaccine strain, including the following four components: (i) deletion of virulence genes *actA* and *inlB*; (ii) *lox* sites flanking essential genes; (iii) inducible *P*_actA_-*cre* at the *actA* locus; (iv) vaccine antigen cassette integrated at *inlB*, *tRNA*^Arg^, or *comK* loci. (b) Primer pairs were designed to amplify the native configuration of the gyrase region (primers 1 and 2) and the Cre-mediated excision product (primers 1 and 3). DC2.4 cells were infected with LADD or Lm-RIID, and infected cell lysates at 0, 3, or 16 h postinfection were used as templates for PCRs with primers 1 and 2 (native, N) or primers 3 and 4 (excised, E). White lines on the PCR gel indicate where lanes were digitally rearranged to group data by LADD and Lm-RIID platforms.

To construct a vaccine with short-term viability that is still capable of robust antigen expression, we developed a suicidal L. monocytogenes strain that deletes essential genes upon reaching the intracellular milieu. Initially, LADD (Δ*actA* Δ*inlB* strains) was engineered with *lox* sites flanking three sets of genes including or adjacent to the replication origin (Fig. 1a; see also Fig. S1 in the supplemental material). The Cre recombinase gene was inserted at the *actA* locus under the control of the *actA* promoter, which is not expressed in broth but induced when L. monocytogenes reaches the host cytosol (25).

Genes near or encompassing the replication origin are essential due to roles in DNA replication, repair, and recombination (26–28). To test whether gene excision decreases viability, growth of floxed L. monocytogenes strains with or without Cre was compared in broth and during infection of murine dendritic-like DC2.4 cells. Strains with (e.g., BH3618) or without (e.g., BH3210) Cre grew identically in broth (Fig. S2a, c, and e). While strains without Cre increased CFU by two logs in DC2.4 cells over 7 h, Cre^+^ L. monocytogenes CFU decreased by four logs (Fig. S2b, d, and f). These data demonstrate that suicidal L. monocytogenes loses viability following infection of host cells, likely as a result of the deletion of essential genes.

### Gene excision by suicidal L. monocytogenes.

We hypothesized that Cre induction leads to excision of floxed genes, resulting in bacteria unable to replicate their DNA. To measure excision, we focused on BH3618 that contains floxed *yaaA*, *recF*, *gyrB*, and *gyrA* (here referred to as Lm-RIID, for Listeria monocytogenes recombinase-induced intracellular death). Strains with a floxed origin (29, 30) formed chains inside cells, which generally correlates with poor immunogenicity (P. Lauer, unpublished observations), so these were not pursued further. LADD and Lm-RIID were used to infect DC2.4 cells, and PCR was performed to distinguish between the intact *yaaA-recF-gyrB-gyrA* locus and the excised DNA product (Fig. 1b). The excision product was detected soon after Lm-RIID infection, increased over time, and inversely correlated with native product levels. In contrast, LADD samples exclusively contained the native product throughout the infection. These results demonstrate that Lm-RIID excises essential genes after accessing host cytosol, consistent with decreased CFU numbers in DC2.4 cells.

### Lm-RIID loses intracellular activity by 9 hpi.

Numbers of bacterial CFU following infection were reduced as early as 3 h postinfection (hpi). However, when growth was monitored by microscopy, LADD and Lm-RIID numbers were similar, at least up to 5 hpi (Fig. S3a and b). These results were surprising, as Lm-RIID CFU clearly decreased compared with those of LADD. Importantly, CFU enumeration requires further rounds of replication for colony formation while imaging captures replication directly.

**FIG 2:**
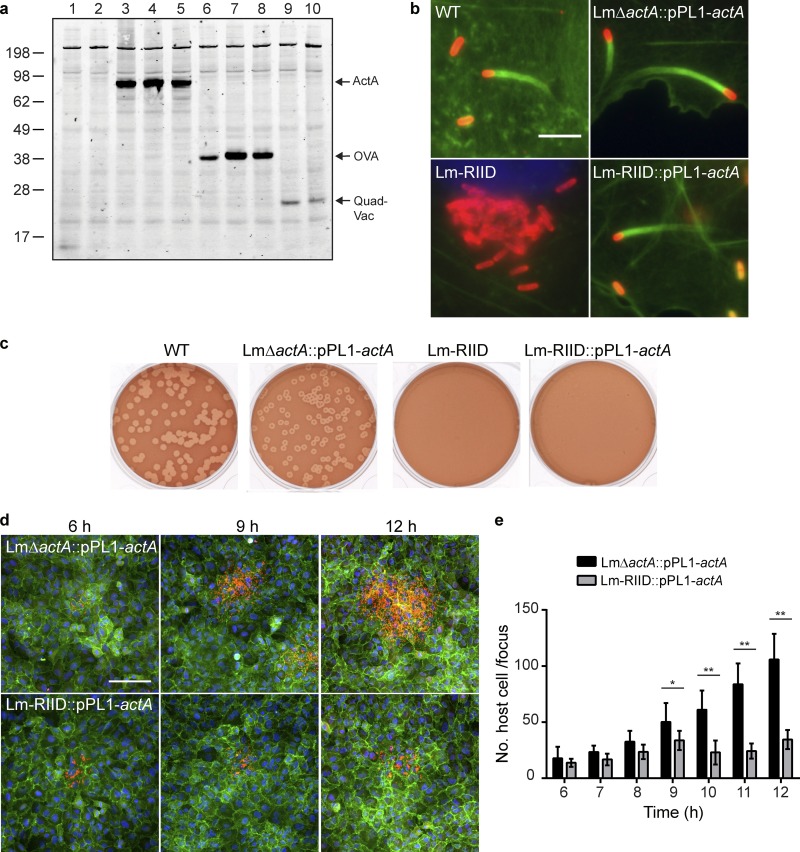
Lm-RIID stops spreading from cell to cell at 9 h postinfection. (a) Intracellular Western blot of infected cell lysates from 8-h DC2.4 infections. A polyclonal antibody specific to the mature amino terminus of the ActA protein was used to detect ActA and ActA fusion proteins. Lanes 1 and 2, empty vector negative controls of LADD and Lm-RIID; 3 to 5, WT L. monocytogenes, Lm-*act*::pPL1-*actA*, and Lm-RIID::pPL1-*actA* strains; 6, OVA in LADD; 7 and 8, AH1/A5-OVA fusion protein in LADD and Lm-RIID; 9 and 10, QuadVac fusion protein in KBMA and Lm-RIID platforms. (b) Fluorescence images of actin tails formed by WT, Lm-*act*::pPL1-*actA*, Lm-RIID, and Lm-RIID::pPL1-*actA* strains. L. monocytogenes organisms were stained with α-L. monocytogenes O-antigen antibody (red), host actin filaments with Alexa Fluor-488 phalloidin (green), and DNA with DAPI (blue). Scale bar, 5 μm. (c) Representative images of plaque assays with L2 cells at 72 h postinfection. (d) Fluorescence images of infectious foci formed by Lm-*act*::pPL1-*actA* and Lm-RIID::pPL1-*actA* strains in A549 cells. L. monocytogenes organisms were stained with α-L. monocytogenes O-antigen antibody (red), host cell membranes with α-β-catenin antibody (green), and DNA with DAPI (blue). Scale bar, 100 μm. (e) The number of host cells per infectious focus following infection of A549 cells with the Lm-*act*::pPL1-*actA* or Lm-RIID::pPL1-*actA* strain. Asterisks denote Lm-RIID::pPL1-*actA* foci significantly different from Lm-*act*::pPL1-*actA* foci (*, *P* < 0.1; **, *P* < 0.0001; both by two-way analysis of variance [ANOVA]).

To investigate the functional capabilities of Lm-RIID, we used a plaque assay, which measures growth and cell-to-cell spread and requires 3 days and multiple infection cycles to occur. Because plaque formation requires ActA, *actA* was integrated site specifically at the *comK* locus of Lm-RIID (31). ActA expression was similar following infection with WT L. monocytogenes, an *actA* deletion strain (LmΔ*actA*::pPL1-*actA* strain), or an Lm-RIID strain complemented at the *comK* locus (Lm-RIID::pPL1-*actA* strain) (Fig. 2a, lanes 3 to 5), and ActA^+^ strains formed actin tails in host cells (Fig. 2b). Three days following infection, WT and LmΔ*actA*::pPL1-*actA* strains formed plaques but Lm-RIID did not, consistent with the presence of ActA and actin tail formation in the first two strains (Fig. 2c). Importantly, the Lm-RIID::pPL1-*actA* strain was unable to form plaques despite its ability to form actin tails. These data demonstrate that although growth of Lm-RIID cells is visible by microscopy at 5 hpi and they form actin tails at 6 hpi, they are unable to spread in a cell monolayer over 3 days of infection.

To more precisely determine when Lm-RIID bacteria are affected by chromosomal excision, cell-to-cell spread was measured by an infectious focus assay (32). Importantly, growth of L. monocytogenes strains in A549 cells was similar to that in DC2.4 cells (Fig. S3c). WT L. monocytogenes cells form foci of 24 host cells per focus (HC/F) at 6 hpi, and these grow to 120 HC/F by 12 hpi (Fig. S4). LmΔ*actA*::pPL1-*actA* cells form foci of size similar to that of WT L. monocytogenes (Fig. 2d and Fig. S4). In contrast, Lm-RIID::pPL1-*actA* foci plateau at 34 HC/F at 9 hpi with no further increase in size, demonstrating that Lm-RIID cells stop spreading at this time (Fig. 2d and e).

### Accelerated clearance of Lm-RIID in WT and immunocompromised mice.

**FIG 3:**
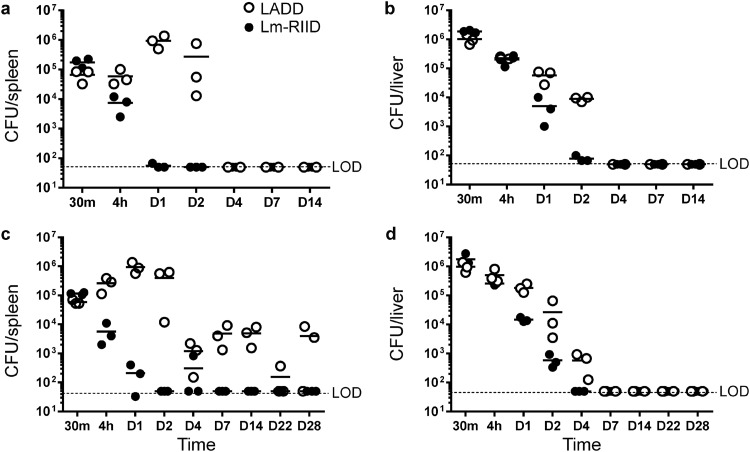
Lm-RIID is cleared from WT and immunocompromised mice faster than LADD. WT (a and b) or CD-1 nude (nu/nu) (c and d) mice were vaccinated i.v. with 5 × 10^6^ CFU of LADD (open circles) or Lm-RIID (solid circles). After vaccination (30 min, 4 h, and 1, 2, 4, 7, 14, 22, and 28 days), spleens and livers were homogenized, dilutions were plated on BHI medium, and CFU per organ were enumerated. LOD, limit of detection.

Viable Lm-RIID was not recoverable from host cells at 7 hpi. However, whether this strain replicated in animals was unknown. To measure replication *in vivo*, CD-1 mice were vaccinated intravenously (i.v.) with LADD or Lm-RIID, and CFU in spleen and liver were enumerated over time. Lm-RIID was cleared faster than LADD, with no detectable CFU by day 1 in spleen and day 2 in liver (Fig. 3a and b). LADD was not cleared until 4 days postvaccination, consistent with previous observations (5). We hypothesized that *in vivo* clearance of Lm-RIID should be independent of the host immune status. To test this idea, clearance was measured in CD-1 nude mice that lack T cells. LADD clearance in nude mice took longer than that of WT mice (28 versus 4 days in spleen; Fig. 3c and d). In contrast, Lm-RIID was cleared with similar kinetics in nude and WT mice, demonstrating that Lm-RIID organisms drive their own accelerated clearance independent of the host.

### Decreased virulence of Lm-RIID::pPL1-*actA* strain.

The increased clearance rate suggests Lm-RIID is less virulent than LADD. To determine whether Lm-RIID virulence is reduced, median lethality (i.e., LD_50_) was measured in mice (Table S2). LADD itself is highly attenuated, and Lm-RIID displayed a similarly high level of attenuation (LD_50_, 1 × 10^8^) compared to that of WT L. monocytogenes (1 × 10^5^). Cre expression alone did not further attenuate LADD (strain BH3099, LD_50_ of ∼1 × 10^8^). However, complementation with *actA* revealed an additional level of attenuation for Lm-RIID that would not be appreciated from the strain lacking *actA* due to an upper limit of ∼1 × 10^8^ for LD_50_ measurements. While LADD complemented for *actA* (LADD::pPL1-*actA* strain) exhibited an LD_50_ identical to that of WT L. monocytogenes (1 × 10^5^), the LD_50_ of the Lm-RIID::pPL1-*actA* strain was 3 logs less (LD_50_ of 1 × 10^8^).

### Lm-RIID induces a robust immune response.

**FIG 4:**
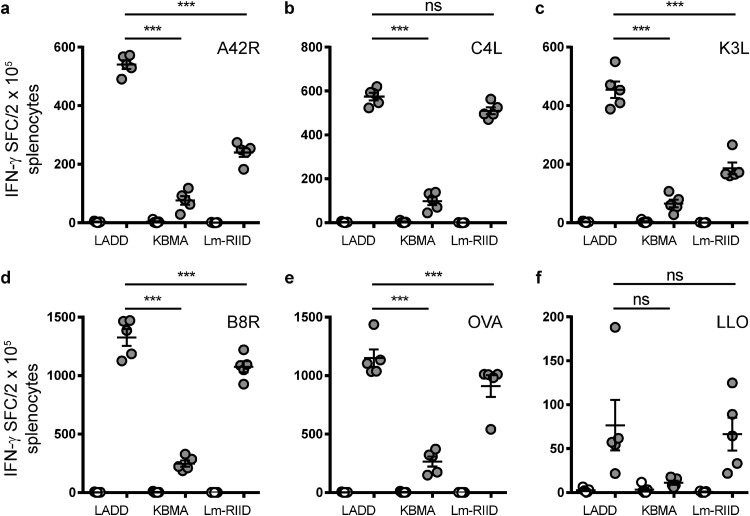
Lm-RIID induces robust immune responses to heterologous vaccinia virus antigen. C57BL/6 mice were injected (5 × 10^6^ CFU) i.v. twice 5 weeks apart with LADD, KBMA, or Lm-RIID containing an antigen expression cassette that includes four vaccinia CD8^+^ T cell epitopes and the OVA_257–264_ (SIINFEKL) epitope. Splenic T cell responses were measured by IFN-γ ELISPOT assay. Unstimulated responses (open circles) and stimulated responses (gray circles) are shown for A42R (a), C4L (b), K3L (c), B8R (d), OVA_257–264_ (e), and LLO_296–304_ (f). The L. monocytogenes-specific CD8^+^ T cell epitope LLO_296–304_ served as a positive control. Significance was calculated using a two-way ANOVA: ***, *P* < 0.0001; ns, not significant.

Although accelerated clearance and reduced virulence may increase vaccine safety, these changes may also impact immunogenicity. To test the potency of Lm-RIID, we compared immunogenicity to LADD and KBMA strains (3). KBMA is a version of LADD that is inactivated by cross-linking DNA, which prevents replication. To measure immune responses, an antigen expression cassette composed of 4 vaccinia CD8 epitopes as well as the OVA_257–264_ epitope (SIINFEKL; the entire cassette was termed QuadVac) was integrated at the *inlB* locus of Lm-RIID, KBMA (6), and LADD strains (Fig. 1a). QuadVac was expressed at similar levels from Lm-RIID and KBMA platforms following DC2.4 infection (Fig. 2a, lanes 9 and 10). Enzyme-linked immunosorbent spot (ELISPOT) assay measurement of splenocytes 7 days postimmunization revealed that KBMA elicited 4- to 7-fold-reduced immune responses, whereas Lm-RIID induced responses that were either similar to (C4L_125–132_ and LLO_296–304_) or modestly reduced (1.2-fold decrease for B8R_20–27_ and OVA_257–264_ and 2- to 2.5-fold decrease for A42R_88–96_ and K3L_6–15_) relative to those of LADD (Fig. 4). These results indicate that Lm-RIID maintains the ability to induce robust responses to foreign antigens.

### Lm-RIID provides protective immunity in bacterial and viral challenge models.

**FIG 5:**
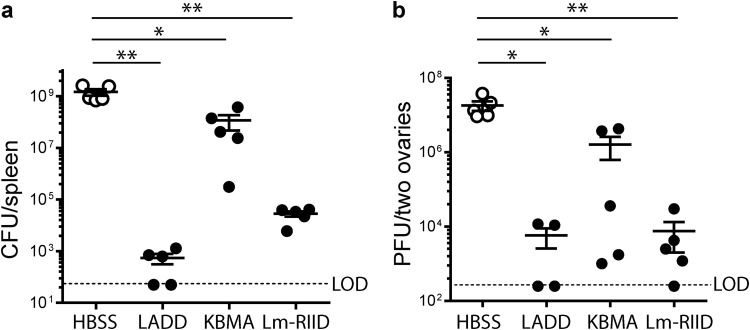
Lm-RIID vaccination protects against virulent WT L. monocytogenes and vaccinia virus challenge. (a) C57BL/6 mice were vaccinated once with a LADD, KBMA, or Lm-RIID strain that expresses the QuadVac cassette. Mice were challenged 6 weeks later with twice the median lethal dose (LD_50_) of WT L. monocytogenes. (b) C57BL/6 mice were vaccinated twice 30 days apart with a LADD, KBMA, or Lm-RIID QuadVac strain. Mice were challenged 7 weeks later with WT vaccinia virus. Five days after challenge, PFU were enumerated from ovaries of infected mice. Significance was determined using a Student's *t* test: *, *P* < 0.05; **, *P* < 0.01.

A hallmark of L. monocytogenes immunity is the induction of life-long protection against virulent L. monocytogenes challenge after a single vaccination, a measure of priming functional T cells. To determine if Lm-RIID can protect against a lethal L. monocytogenes challenge, mice were immunized with LADD, KBMA, or Lm-RIID and challenged 6 weeks later with twice the LD_50_ of WT L. monocytogenes. Lm-RIID provided 5 logs of protection, nearly the level of LADD (6 logs of protection; Fig. 5a), while KBMA provided 1 log of protection. To assess immune response quality in a heterologous virulence challenge, mice were vaccinated twice with each QuadVac-expressing strain and then challenged with vaccinia virus. In this model, KBMA provided just 1 log of protection, while both LADD and Lm-RIID provided greater than 3 logs of protection (Fig. 5b), indicating that Lm-RIID protects against bacterial and viral challenge, similar to LADD.

### Lm-RIID confers therapeutic efficacy in a tumor challenge model.

**FIG 6:**
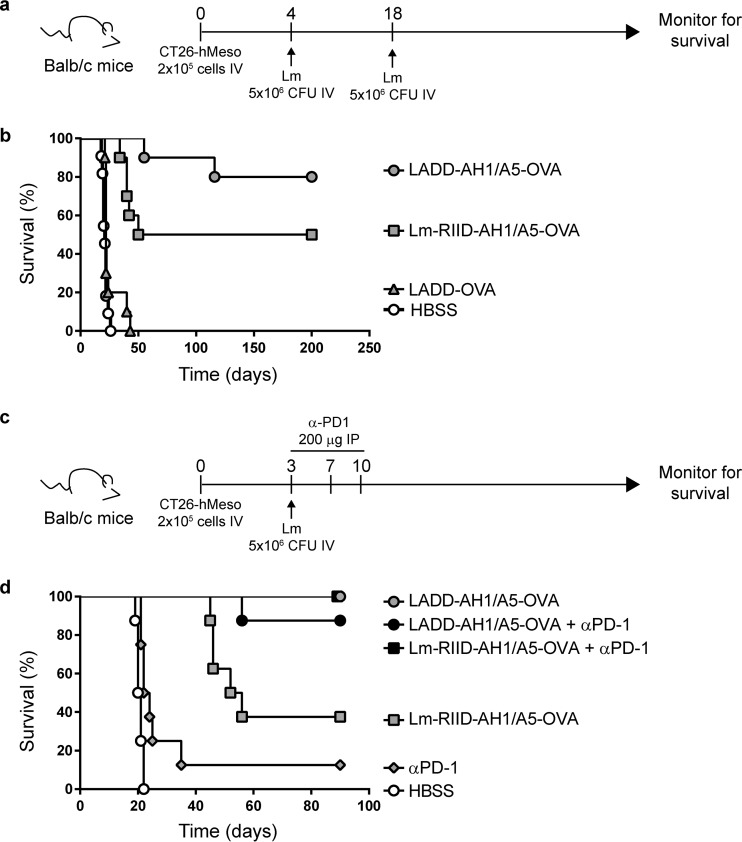
Lm-RIID is partially effective alone and fully effective in combination with PD1 checkpoint blockade at curing mice with CT26 lung metastases. (a) Schematic of the CT26 lung metastasis model and L. monocytogenes treatment regimen. (b) BALB/c mice were challenged i.v. with 2 × 10^5^ CT26 tumor cells and vaccinated 4 and 18 days later with 5 × 10^6^ CFU of LADD-OVA, LADD-AH1/A5-OVA, or Lm-RIID-AH1/A5-OVA and then monitored for survival. (c) Schematic of the CT26 lung metastasis model and L. monocytogenes and α-PD1 treatment regimen. IP, intraperitoneal. (d) BALB/c mice were challenged i.v. with 2 × 10^5^ CT26 tumor cells and vaccinated 3 days later with 5 × 10^6^ CFU of LADD-OVA, LADD-AH1/A5-OVA, or Lm-RIID-AH1/A5-OVA. Two additional groups vaccinated with LADD-AH1/A5-OVA or Lm-RIID-AH1/A5-OVA also received α-PD1 (200 μg) on days 3, 7, and 10 postimplantation. Survival is plotted for each group, and groups are identified in the panel.

To evaluate efficacy in a therapeutic CT26 lung metastasis model (Fig. 6a), LADD and Lm-RIID were engineered to express the T cell epitope AH1-A5 from the tumor rejection antigen gp70 (33, 34) fused to ovalbumin (OVA) (LADD-AH1-A5-OVA and Lm-RIID-AH1-A5-OVA). Both strains expressed similar levels of AH1-A5-OVA protein (Fig. 2a, lanes 7 and 8). LADD expressing OVA alone was used as a negative control (LADD-OVA). LADD-AH1-A5-OVA prolongs survival in this model compared to Hank’s balanced salt solution (HBSS) and LADD-OVA controls (*P* < 0.0001) (5). Lm-RIID-AH1-A5-vaccinated mice also exhibited prolongation of life, with 50% long-term survivors (Fig. 6b), demonstrating that Lm-RIID displays significant therapeutic efficacy in this model.

### Combination therapy with Lm-RIID and α-PD-1 is as effective as LADD treatment.

The checkpoint inhibitor antibody α-programmed cell death 1 (α-PD-1) mediates profound clinical responses in patients with cancer (35), particularly in malignancies with high mutational burden (36). α-PD-1 treatment reinvigorates CD8 T cells by blocking inhibitory signaling, although one prerequisite for checkpoint therapy responsiveness is an inflamed tumor microenvironment (TME) (37). Checkpoint therapy could synergize with treatments that induce T cell responses and inflame the TME, such as LADD vaccination (12, 14, 15). To test this idea, LADD, Lm-RIID, and α-PD-1 were tested in the CT26 lung metastasis model, similar to the monotherapy experiment, but with or without α-PD-1 (Fig. 6a and c). Monotherapies resulted in low (1/8 survivor, α-PD-1) or modest (3/8 survivors, Lm-RIID) survival benefit (Fig. 6d). However, the combination of Lm-RIID and α-PD-1 increased survival to 100%, the same as that seen for LADD monotherapy. These data indicate that Lm-RIID synergizes with checkpoint inhibition to cure CT26 lung metastases and demonstrate that Lm-RIID can be as efficacious as LADD for cancer treatment.

## DISCUSSION

Effective vaccinology requires a delicate balance between safety (attenuation) and immune potency. Diverse approaches have been developed among recombinant bacterial and viral platforms that prevent pathogenicity but preserve the capacity to target antigen-presenting cells, activate innate immunity, and present antigens on major histocompatibility complex class I and class II molecules. The ideal vaccine strain would be that which is the most highly attenuated yet elicits the strongest functional immune responses. We have previously described the development of the LADD platform, which has been tested extensively in patients with diverse malignancies (5, 14, 15). Here, we describe Lm-RIID, which contains an additional layer of safety through a genetic program that catalyzes its own destruction following antigen expression and presentation.

Lm-RIID is more attenuated than LADD, as evidenced by its increased clearance rate from mice and an increased LD_50_ in an *actA*^+^ background. Furthermore, the nearly irreversible nature of Cre with mutant *lox* sites (38) in Lm-RIID ensures that reversion is highly unlikely. Therefore, Lm-RIID is an attractive alternative to LADD. Although Lm-RIID induced immune responses similar to those of LADD for some epitopes, other responses were reduced as much as 2.5-fold, indicating that the higher degree of attenuation dampens immunogenicity. Despite this reduction, Lm-RIID protects animals against L. monocytogenes and vaccinia challenges, demonstrating that its immunogenicity is sufficient for protection. The antitumor efficacy of Lm-RIID with α-PD1 further indicates that Lm-RIID is potent enough to generate effective responses. The quality of CD8^+^ T cells is also a key factor in driving effective immune responses, and additional experiments are required to determine whether the quality of T cells induced by Lm-RIID and LADD differs. Furthermore, how these preclinical results will translate to humans remains to be determined.

Excision of essential genes reduces Lm-RIID viability in host cells, which is apparent by 3 hpi when measuring CFU. However, direct observations by microscopy indicated that Lm-RIID organisms continue to divide at least through 5 hpi. Measuring infectious foci of ActA^+^ strains revealed that Lm-RIID stops spreading around 9 hpi. This is consistent with measurements of protein expression, which were similar for LADD and Lm-RIID at 8 hpi. A delay in the loss of Lm-RIID viability is not surprising and may reflect bacteria that have undergone chromosomal excision yet maintain YaaA, RecF, GyrB, and/or GyrA activity prior to death, presumably as these essential proteins are diluted by replication. Increasing the half-life of one or more of these proteins could extend Lm-RIID survival.

KBMA is an attenuated version of LADD that is defective for replication under any condition (3). *uvrAB* deletion prevents cross-link repair yet allows for metabolic activity and escape from the phagolysosome following host cell invasion. ActA^+^ KBMA recruits host actin but cannot undergo actin-based motility, an effect that was observed as early as 5 hpi, earlier than when Lm-RIID stops spreading at 9 hpi. Consistent with an earlier growth defect, KBMA had a greater reduction in immunogenicity than Lm-RIID (5- versus 2.5-fold), suggesting that the longer duration of growth is necessary for maximum immune responses. Further Lm-RIID modification or, as discussed below, combining Lm-RIID with other immunotherapies may boost responses.

Lm-RIID and KBMA are similarly efficacious as monotherapies in mouse tumor models (40 to 50% survival for both). Checkpoint inhibition has not been tested with KBMA, raising the question of whether this would similarly enhance efficacy. Studies examining the effect of checkpoint inhibition on LADD immune responses suggest that α-PD1 treatment prolongs the activation of LADD-induced cytotoxic T cells (W. Deng, unpublished data). Other immunomodulatory therapies, such as α-CTLA4, α-OX40, and α-GITR, also synergize with LADD in preclinical models (39 and M. Leong, C. Rae, and W. Deng, unpublished data), and the effect of these on Lm-RIID efficacy will be important to evaluate as well. The ability of different immunomodulatory antibodies to alter Lm-RIID responses could reveal keys to the mechanism of action of this vaccine.

A common strategy for attenuation of live bacterial vaccines is through inhibition of intracellular replication. Some notable examples include a Salmonella enterica serotype Typhi *galE* mutant, which takes up galactose for cell wall incorporation that enables immunogenicity and protection but also leads to the accumulation of toxic levels of galactose metabolites (40). Francisella novicida strains were engineered to express psychrophilic essential genes (41). These strains displayed temperature-sensitive (ts) growth, with normal growth at 30 to 35°C but no growth in host cells at 37°C, and they elicited protective immunity in mice. Lm-RIID is similar to these ts strains in that essential proteins are depleted to eliminate vaccine following antigen expression; however, it takes advantage of genetic removal of genes rather than ts proteins. The Lm-RIID strategy may enable a longer half-life, which could enhance antigen delivery and immune responses.

L. monocytogenes vaccines have shown promising results both preclinically and in clinical trials, yet there are limitations on which patients can be treated with attenuated L. monocytogenes. Lm-RIID represents an important step toward the development of L. monocytogenes vaccines that can be administered to a broader population of patients and used for the prophylactic treatment of infectious diseases. The synergy of Lm-RIID with checkpoint inhibition is particularly promising in light of the expanding use of checkpoint therapy and suggests an obvious immunotherapy combination to explore further.

## MATERIALS AND METHODS

### Bacterial strain construction.

Strains used in this study are listed in Table S1 in the supplemental material. Allelic exchange (including deletions and *lox*, *frt*, Cre, FLP, and antigen expression cassette insertions) was performed with *oriT*-containing derivatives of pKSV7 as previously described (31, 42). Site-specific integration at *comK* and *tRNA*^Arg^ was performed with the pPL1 and pPL2 integration vectors as previously described (31). The Cre and FLP recombinase coding sequences were optimized for *Listeria* expression, synthesized (ATUM, Newark, CA), and cloned immediately downstream of the *actA* promoter. For direct comparisons to KBMA, some suicidal strains were constructed in the KBMA background to allow for photochemical inactivation with S-59 (3). Complementation of ActA was achieved with pPL25 as previously described (31). The QuadVac expression cassette expresses four CD8^+^ T cell epitopes fused to the amino terminus of ActA (6). The gp70 epitope strains AH1 (WT; SPSYVYHQF) and AH1/A5 (APL; SPSYAYHQF) were based on a previously described ActA-(epitope)-OVA fusion strategy (43) and were constructed by epitope site-specific integration at *tRNA*^Arg^ with pPL2. Strains were verified by PCR analysis and sequencing.

### *In vitro* growth curves.

Overnight brain heart infusion (BHI) cultures were diluted 1:100, aliquoted (150 μl per well) into a 96-well, flat-bottom plate, and monitored for growth using a VersaMax microplate reader (Molecular Devices, Sunnyvale, CA).

### Intracellular growth curves.

Murine dendritic DC2.4 cells (44) were maintained in RPMI 1640 (Thermo Fisher Scientific, Waltham, MA) supplemented with 10% heat-inactivated fetal bovine serum (FBS; HyClone, GE Healthcare, Pittsburgh, PA), 23.8 mM sodium bicarbonate (Sigma, Saint Louis, MO), 1× nonessential amino acids (Cellgro; Corning, Tewksbury, MA), 2 mM l-glutamine (Cellgro), 10 mM HEPES buffer (Gibco), 1 mM sodium pyruvate (Sigma), and 50 μM β-mercaptoethanol (Sigma). Human lung carcinoma A549 cells (ATCC, Manassas, VA) were maintained in Ham’s F-12K medium (Gibco) with 10% heat-inactivated FBS (HyClone). Growth curves in both cell types were performed in 24-well tissue culture plates (Costar 3524; Corning, NY). For DC2.4 studies, cells (2 × 10^5^) were seeded in each well and infected at a multiplicity of infection (MOI) of 20. For A549 studies, cells (1.5 × 10^5^) were seeded in each well and infected at an MOI of 50. L. monocytogenes strains were grown overnight in BHI at 30°C without agitation and then diluted into cell culture media such that the total CFU for the desired MOI was in a 0.5-ml volume. Cells were infected for 1 h and washed with 1 ml phosphate-buffered saline (PBS; HyClone), and then cell culture medium containing gentamicin (50 μg/ml) was added to prevent growth of extracellular bacteria. Bacteria were harvested at increasing times postinfection by aspirating the media, washing cell monolayers (1 ml PBS), and lysing the cell monolayer hypotonically with sterile water (1 ml). Serial dilutions were plated on BHI agar plates supplemented with streptomycin (200 μg/ml; Teknova, Hollister, CA) and incubated overnight at 37°C, and colonies were counted to determine the number of CFU/well. Each strain was measured in triplicate, and the means with the standard deviations are reported.

### PCR chromosomal excision assay.

DC2.4 cells (1 × 10^6^) were seeded in 12-well tissue culture plates and incubated overnight. The following day, cells were infected at an MOI of 25 for 1 h and rinsed twice with PBS, and then cell culture medium containing gentamicin (60 μg/ml) was added. Infected lysates were harvested at increasing times postinfection by aspirating culture media, rinsing once with PBS, and lysing cells in water by repeated pipetting and scraping. Three wells for each strain and time point were pooled, PBS was added to a final concentration of 1×, and genomic DNA was isolated using a MasterPure Gram-positive DNA purification kit (Epicentre, Middleton, WI) according to the manufacturer’s instructions. PCRs using genomic DNA as the template and combinations of primer 1 (TCGTAATCGTGGTGGTATGGGT), primer 2 (AAAACTACTGCTATAAACAGA), and primer 3 (TCCACCAGTTGAAACTACATCA) were performed with Phusion polymerase (NEB, Ipswich, MA) on a Bio-Rad T100 thermal cycler (Hercules, CA).

### Intracellular Western blotting.

DC2.4 cells (3 × 10^5^) were seeded in each well of a 12-well tissue culture plate and incubated overnight. Cells were infected at an MOI of 10 for 1 h and rinsed with PBS, and RPMI complete medium containing 50 μg/ml gentamicin was added. Cells were cultured seven additional hours before washing with PBS (1 ml) and lysing cells in 1× lithium dodecyl sulfate (LDS) buffer (150 μl of a mix of 4× LDS buffer [Life Technologies, Grand Island NY], Tris-EDTA buffer [Fisher Scientific], and sample reducing agent [Life Technologies]). Cell lysates were incubated at 95°C for 10 min, and aliquots (20 μl) were run on 4 to 12% Bis-Tris PAGE gels in 1× morpholineethanesulfonic acid buffer (Invitrogen) and transferred to nitrocellulose membranes for detection. Membranes were blocked for 1 h in Odyssey blocking buffer (Li-Cor, Lincoln, NE), and heterologous antigens were detected with a polyclonal rabbit antibody (1:4,000) that recognizes the mature 18-amino-acid N terminus of ActA. Labeled goat anti-rabbit (IRDye 680RD; Odyssey) secondary antibody was used at a 1:10,000 dilution. Antibodies were diluted in Odyssey blocking buffer with 0.2% Tween 20. Membranes were incubated with primary antibody overnight at 4°C, washed three times for 5 min each wash with PBS containing 0.1% Tween, and then incubated with secondary antibody for 1 h at room temperature. Membranes were washed a further four times, the last wash with PBS only, and then scanned using a Li-Cor Odyssey system.

### Fluorescence microscopy.

For fixed imaging of bacterial replication, actin tails, and infectious foci, DC2.4 cells (2 × 10^5^), Cos7 cells (5 × 10^4^), or A549 cells (4.5 × 10^5^) were seeded onto coverslips in 24-well tissue culture plates and infected for 1 h. Cos7 and A549 infections included an initial centrifugation step (200 × *g* for 5 min). After 1 h, monolayers were washed with PBS (1 ml), cell culture medium with gentamicin (50 μg/ml) was added, and coverslips were fixed with 4% formaldehyde (Polysciences) in PBS for 10 min at the times indicated in the text. Cos7 cells were maintained in Dulbecco’s modified Eagle’s medium (DMEM; Gibco) and A549 cells were maintained in Ham’s F-12K medium (Gibco), both with 10% heat-inactivated FBS (HyClone). Immunofluorescence staining was performed by first permeabilizing and blocking coverslips for 30 min at room temperature (2% bovine serum albumin [BSA] and 0.1% Triton X-100 in PBS; BSA-PBSTX), followed by incubation with primary antibody(ies) for 30 min. Coverslips were washed (3×, PBS), incubated with secondary antibody(ies) for 30 min, washed again (3×, PBS), and then mounted onto glass slides with ProLong diamond antifade mountant (Life Technologies). All antibodies were diluted in BSA-PBSTX. L. monocytogenes was detected using an α-*Listeria* O antigen polyclonal rabbit antibody (1:200; BD Biosciences, Franklin Lakes, NJ) and Alexa Fluor 594- or Alexa Fluor 488-conjugated anti-rabbit secondary antibody (1:200; Invitrogen). Cells and actin were visualized by including Alexa Fluor 488- or Alexa Fluor 594-conjugated phalloidin (1:250; Invitrogen) and 4′,6-diamidino-2-phenylindole (DAPI) (Fisher Scientific) in the secondary antibody step. For A549 staining, cell membranes were detected with an α-β-catenin antibody (1:250; BD Biosciences) and an Alexa Fluor 488-conjugated α-mouse secondary antibody (1:250; Invitrogen).

Imaging was performed with an inverted Nikon TiE microscope, and images were captured with an Andor Zyla sCMOS camera controlled by NIS Elements software (Nikon, Melville, NY). Bacterial replication and actin tail images were taken with a 60× (1.4 numeric aperture [NA]) PlanApo objective and infectious focus images with a 20× (0.5 NA) Plan Fluor objective. Image analysis was performed in Fiji (45, 46). For microscopy measurements of intracellular bacterial replication, the number of bacteria in individual host cells was counted using the Cell Counter plugin for ImageJ. For each strain and time point, infected host cells in 10 fields of view (FOV) from two coverslips were counted for a minimum of 150 infected host cells per strain and time point. For infectious focus analysis, the number of host cells per focus was counted using the Cell Counter plugin for ImageJ, and a minimum of 12 infectious foci were analyzed for each strain and time point.

### Plaque assay.

Plaque assays with murine L2 fibroblasts were performed as previously described (47). Briefly, L2 cells (1.2 × 10^6^) were seeded onto 6-well plates 1 day before infection. The next day, the optical densities of static, overnight 30°C cultures of L. monocytogenes were normalized, and cultures were washed three times in PBS and allowed to infect L2 monolayers for 1 h. Cells were washed three times, overlaid with 3 ml of 0.7% agar containing 10 μg/ml gentamicin, and incubated at 37°C. Three days later a second overlay of 2 ml of 0.7% agar-gentamicin and 25 μl neutral red (Sigma-Aldrich) was added, plates were incubated overnight, and plaque size was analyzed using ImageJ (http://rsbweb.nih.gov/ij/). At least three wells were analyzed per mutant per experiment.

### Animals.

C57BL/6, BALB/c, CD1, CD1^nu/nu^, and SCID beige mice were obtained from Charles River Laboratories (Wilmington, MA). Mice were handled according to National Institutes of Health guidelines. All animal protocols were approved by the Aduro Biotech Institutional Care and Use Committee. L. monocytogenes vaccinations were given intravenously at the doses indicated in the text.

### *In vivo* growth kinetics.

CD1 or CD1^nu/nu^ mice were immunized with the indicated L. monocytogenes strains (5 × 10^6^ CFU), and the level of infection in each mouse in the liver and spleen was determined by isolating, homogenizing, diluting, and plating for CFU for each organ.

### Median lethality (LD_50_).

Median lethality experiments were performed as previously described (5). Briefly, C57BL/6 mice were immunized with the indicated L. monocytogenes strains at a dose equal to 0.1 LD_50_ and survival was monitored over time.

### ELISPOT assay and peptides.

ELISPOT assays were performed with lymphocytes isolated from whole mouse blood using Lympholyte-mammal (Cedarlane Labs, Burlington, NC) and a murine gamma interferon (IFN-γ) ELISPOT pair (BD Biosciences, San Jose, CA). Cells (2 × 10^5^) were plated in anti-murine IFN-γ-coated ELISPOT plates (Millipore, Billerica, MA) and incubated overnight at 37°C with the indicated peptides or without peptide as a negative control. A42R_88–96_ (YAPVSPIVI), C4L_125–132_ (LNFRFENV), K3L_6–15_ (YSLPNAGDVI), B8R_20–27_ (TSYKFESV) (48), OVA_257–264_ (SIINFEKL), and LLO_190–201_ (NEKYAQAYPNVS) peptides were synthesized by Synthetic Biomolecules (San Diego, CA). Plates were developed using alkaline phosphatase detection reagents (Invitrogen, Carlsbad, CA) and scanned and quantitated using an ImmunoSpot plate reader and software (CTL Ltd., Cleveland, OH).

### WT L. monocytogenes challenge.

Female C57BL/6 mice were vaccinated once with 5 × 10^6^ CFU of each L. monocytogenes strain. Thirty-four days later, mice were challenged with 2× LD_50_ of the WT L. monocytogenes strain DP-L4056 (31). Three days later, spleens and livers were harvested, homogenized, and diluted. Bacterial burden (CFU per organ) was determined by plating dilutions on BHI agar plates containing 200 μg/ml streptomycin.

### Vaccinia challenge.

Female C57BL/6 mice were vaccinated on day 0 and day 29 with 5 × 10^6^ CFU of each L. monocytogenes strain. Mice were challenged 49 days after the second vaccination with WT vaccinia intraperitoneally (1 × 10^6^ PFU). Ovaries were harvested 5 days postchallenge, and plaque assays were performed to determine the level of protection (3).

### Tumor studies.

Female BALB/c mice were implanted intravenously (i.v.) on day 0 with the CT26 tumor cell line engineered to express human mesothelin (2 × 10^5^). For monotherapy studies, mice were vaccinated i.v. with L. monocytogenes strains (5 × 10^6^ CFU) 4 days postimplantation and boosted with the same dose 14 days later. For combination treatment studies, mice were vaccinated i.v. once with L. monocytogenes strains (5 × 10^6^ CFU) 3 days postimplantation. Combination groups also received intraperitoneal injections of α-PD1 (200 μg) on days 3, 7, and 10 postimplantation. HBSS was used as a negative control for all studies. Mice were weighed and monitored daily and were euthanized upon any signs of stress or labored breathing.

## Supplementary Material

Supplemental file 1
