# Supplementary material for: A Potent and Effective Suicidal Listeria Vaccine Platform
Source: Infect Immun. 2019 Jul 23;87(8):e00144-19. doi: 10.1128/IAI.00144-19 (PMC6652770; doi:10.1128/IAI.00144-19)
Supplement: Supplemental file 1 [file IAI.00144-19-s0001.pdf]

Supplemental Information

Supplemental Figures

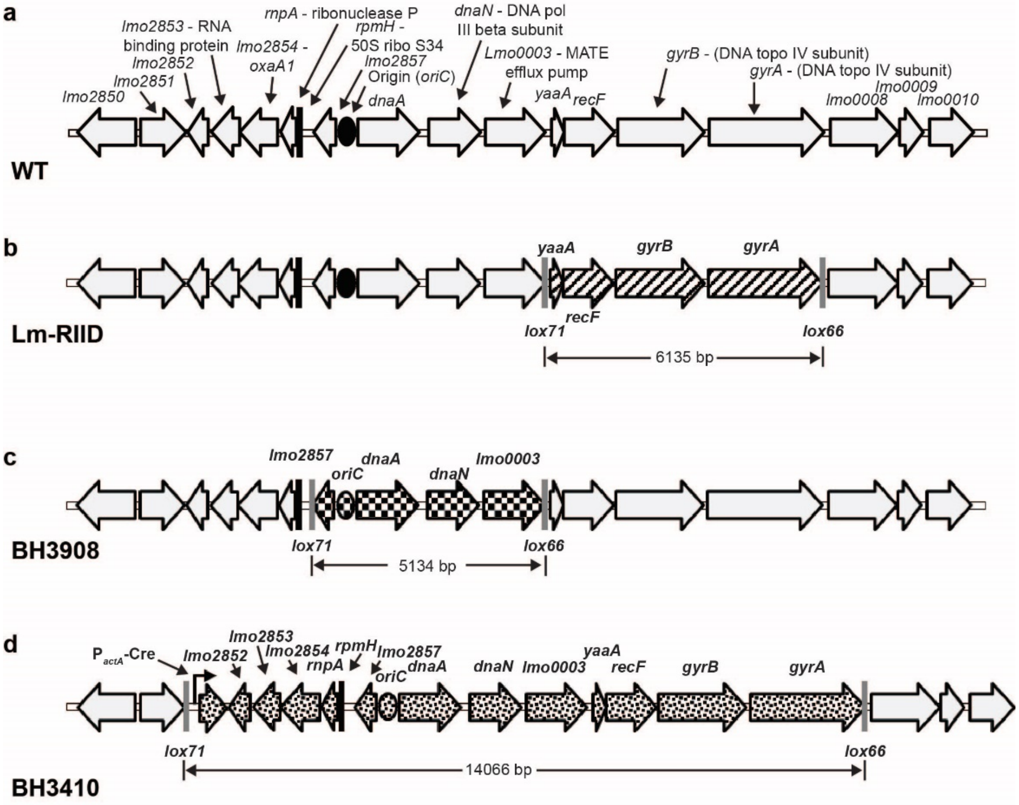

e

Lmo # (EGDe)	LMRG # (10403S)	gene	description
lmo2850	LMRG_02421		major facilitator superfamily transporter
lmo2851	LMRG_02422		hypothetical protein
lmo2852	LMRG_02423		hypothetical protein
lmo2853	LMRG_02424	jag protein	RNA binding protein
lmo2854	LMRG_02425		membrane protein OxaA
lmo2855	LMRG_02426		ribonuclease P
lmo2856	LMRG_02427		50S ribosomal protein L34
lmo2857	LMRG_02428		hypothetical protein
origin		oriC	
lmo0001	LMRG_02429	dnaA	chromosomal replication initiator protein
lmo0002	LMRG_02430	dnaN	DNA polymerase III beta subunit
lmo0003	LMRG_02431		mate efflux pump
lmo0004	LMRG_02432	yaaA	S4 RNA binding protein (replication, recombination, repair)
lmo0005	LMRG_02433	recF	DNA replication and repair
lmo0006	LMRG_02434	gyrB	DNA gyrase subunit B
lmo0007	LMRG_02435	gyrA	DNA gyrase subunit A
lmo0008	LMRG_02436		cardiolipin synthase
lmo0009	LMRG_02437		diamine N-acetyltransferase
lmo0010	LMRG_02439		mevalonate kinase

Supplemental Figure 1. Construction of suicidal LADD strains. Schematics of the Lm chromosomal replication origin region of (a) WT, (b) Lm-RIID (BH3618), (c) BH3908 and (d) BH3410 strains. (e) Lists of the corresponding EGDe and 10403S gene numbers, names and descriptions.

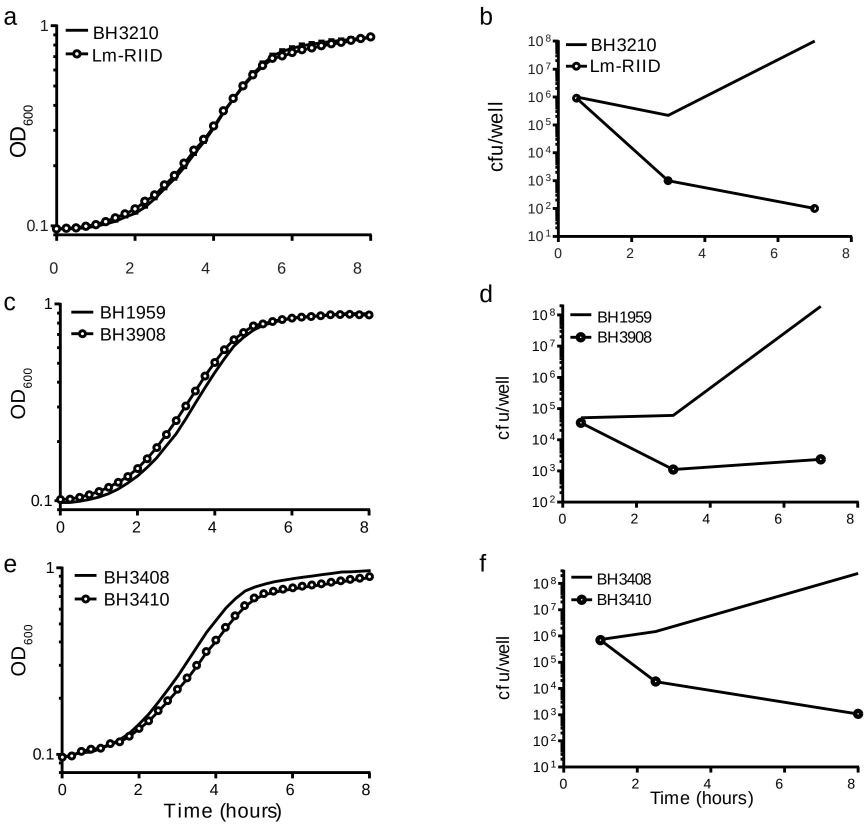

Supplemental Figure 2. Suicidal LADD strains grow similar to parent strains *in vitro* but do not form viable CFUs following recovery from host cells. Growth of suicidal LADD strains (Lm-RIID, BH3908 and BH3410) with matched control strains (BH3210, BH1959 and BH3408) that lack at least one component of the suicide machinery (e.g., *lox* site(s) or *cre*) in (a) Brain Heart Infusion broth or (b) DC2.4 cells.

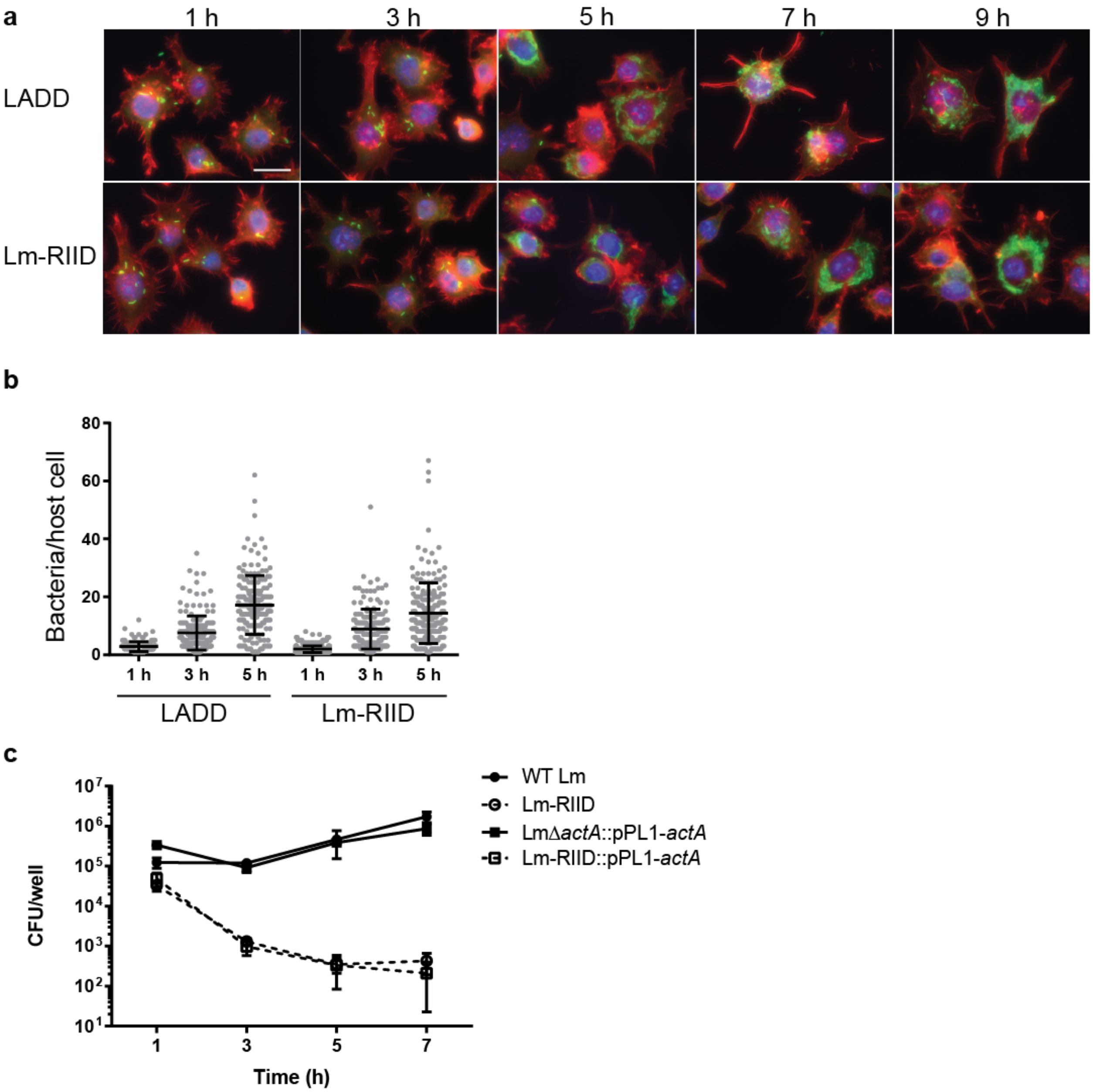

Supplemental Figure 3. (a) Fluorescence images of Lm replication over time. DC2.4 cells were infected with LADD or Lm-RIID for the indicated times, fixed and stained using an α-Lm O-antigen antibody to detect Lm (green), phalloidin to detect host actin filaments (red) and DNA with DAPI (blue). Scale bar, 20 μm. (b) Intracellular replication in DC2.4 cells was measured by counting the number of bacteria per host cell at increasing times post infection. (c) Growth of WT Lm, Lm-RIID, LmΔ*actA*::pPL1-*actA* and Lm-RIID::pPL1-*actA* in A549 cells. The mean CFUs/well from three replicate wells are plotted for each time point with the standard deviation.

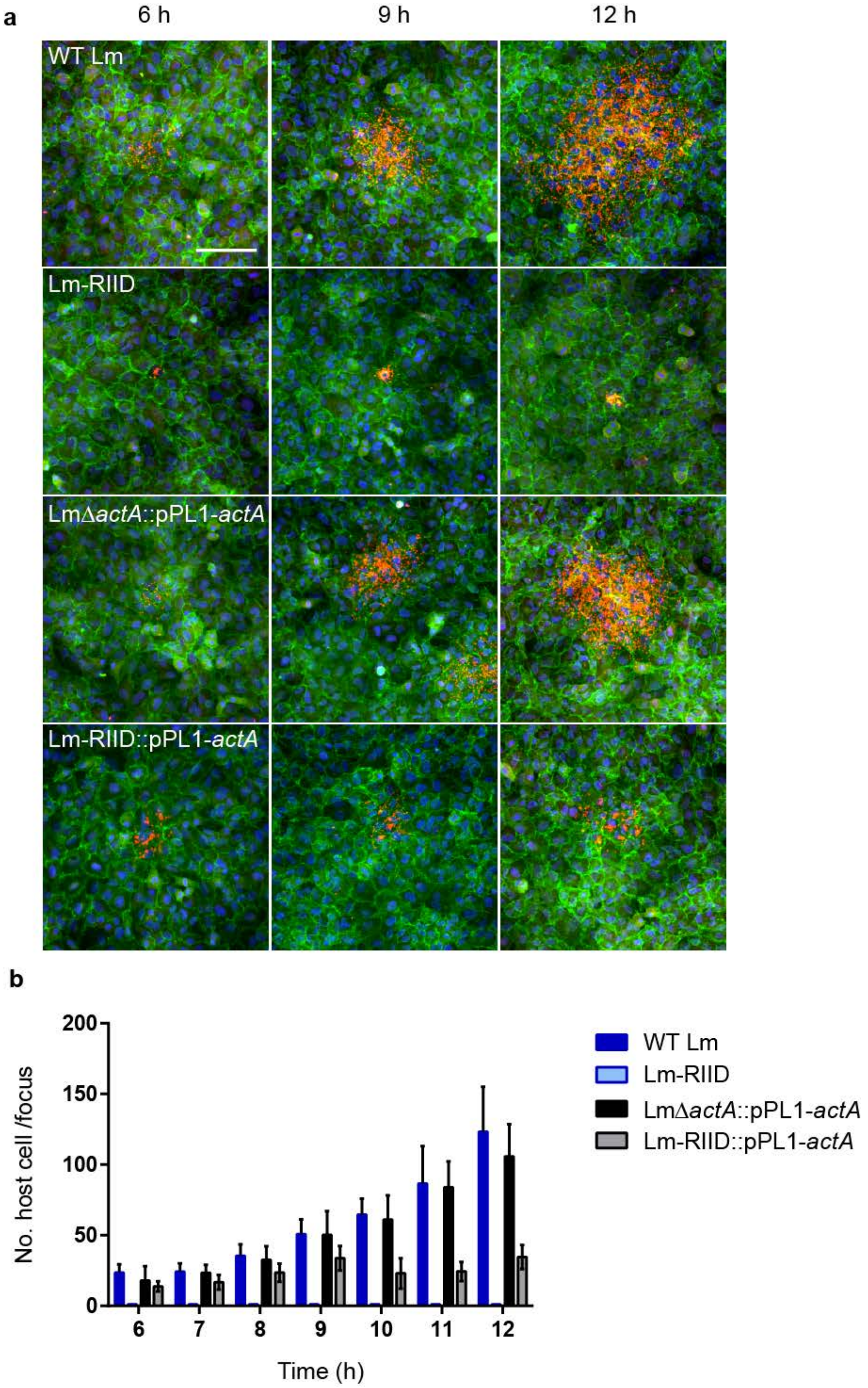

Supplemental Figure 4. (a) Fluorescence images of infectious foci formed by WT Lm, Lm-RIID, LmΔ*actA*::pPL1-*actA* and Lm-RIID::pPL1-*actA* in A549 cells. Lm were stained with α-Lm O-antigen antibody (red), host cell membranes with α-β-catenin antibody (green) and DNA with DAPI (blue). Scale bar, 100 μm. (b) The number of host cells per infectious focus following infection of A549 cells with WT Lm, Lm-RIID, LmΔ*actA*::pPL1-*actA* or Lm-RIID::pPL1-*actA*. Data for LmΔ*actA*::pPL1-*actA* or Lm-RIID::pPL1-*actA* are the same as that shown in Figure 2.

Supplemental Table 1. Bacterial strains used in this study.

Strain	Genotype	Parent strain	Recombinase cassette and locus	Antigen cassette and locus
LADD (Lm11)	Δ*actA* Δ*inlB*	DP-L4056[1]	none	none
KBMA (Lm583)	Δ*actA* Δ*inlB* Δ*uvrAB*	Lm11	none	none
BH3291	Δ*actA* Δ*inlB gyrA-lox66-lmo0008*	Lm11	none	none
BH3339	Δ*actA* Δ*inlB lmo0003-lox71-yaaA gyrA-lox66-lmo0008*	BH3291	none	none
Lm-RIID (BH3618)	Δ*actA* Δ*inlB lmo0003-lox71-yaaA gyrA-lox66-lmo0008*	BH3339	*actA*::P*actA*-Cre	none
KBMA::Quadvac (BH1959)	Δ*actA* Δ*inlB* Δ*uvrAB*	Lm583	none	*inlB*::ActAN100-QuadVac
BH3141	Δ*actA* Δ*inlB* Δ*uvrAB rpmH-lox71-lmo2857*	BH1959	none	*inlB*::ActAN100-QuadVac
BH3901	Δ*actA* Δ*inlB* Δ*uvrAB rpmH-lox71-lmo2857 lmo0003-lox66-yaaA*	BH3141	none	*inlB*::ActAN100-QuadVac
BH3908	Δ*actA* Δ*inlB* Δ*uvrAB rpmH-lox71-lmo2857 lmo0003-lox66-yaaA*	BH3901	*tRNA*^{Arg}::P*actA*-Cre	*inlB*::ActAN100-QuadVac
BH3408	Δ*actA* Δ*inlB lmo2851-lox71*-P*actA*-Cre-*lmo2852*	Lm11	adjacent to *lox71*	none
BH3410	Δ*actA* Δ*inlB lmo2851-lox71-lmo2852 gyrA-lox66-lmo0008*	BH3291	adjacent to *lox71*	none
BH3210	Δ*actA* Δ*inlB lmo0003-lox71-yaaA gyrA-lox66-lmo0008*	BH3141	none	*inlB*::ActAN100-QuadVac
WT Lm (DP-L4056)[1]		10403s[2]	none	none
LmΔ*actA*::pPL1-*actA* (DP-L4077)[1]	Δ*actA*	DP-L4029[1,3]	none	*comK*::pPL1-*actA*
Lm-RIID::pPL1-*actA* (BH3683)	Δ*actA* Δ*inlB lmo0003-lox71-yaaA gyrA-lox66-lmo0008*	BH3618	*actA*::P*actA*-Cre	*comK*::pPL1-*actA*

BH3099	Δ*actA* Δ*inlB* Δ*uvrAB*	BH1959	*tRNA*^{Arg}::P*actA*-Cre	*inlB*::ActAN100-QuadVac
LADD::pPL1-*actA* (BH5104)	Δ*actA* Δ*inlB*	Lm11	none	*comK*::pPL1-*actA*
Lm-RIID::Quadvac (BH3226)	Δ*actA* Δ*inlB lmo0003-lox71-yaaA gyrA-lox66-lmo0008*	BH3210	*tRNA*^{Arg}::P*actA*-Cre	*inlB*::ActAN100-QuadVac
Lm-RIID::pPL1-*actA* (BH5108)	Δ*actA* Δ*inlB lmo0003-lox71-yaaA gyrA-lox66-lmo0008*	BH3618	*actA*::P*actA*-Cre	*comK*::pPL1-*actA*
LADD-AH1/A5-OVA (BH137)	Δ*actA* Δ*inlB*	Lm11	none	*tRNA*^{Arg}::ActAN100-AH1/A5-OVA
Lm-RIID-AH1/A5-OVA (BH3859)	Δ*actA* Δ*inlB lmo0003-lox71-yaaA gyrA-lox66-lmo0008*	BH3816	*actA*::P*actA*-Cre	*tRNA*^{Arg}::ActAN100-AH1/A5-OVA
LADD-OVA (BH892)	Δ*actA* Δ*inlB*	Lm11	none	*tRNA*^{Arg}::ActAN100-OVA

[1], Lauer, et al., 2002

[2], Bishop and Hinrichs, 1987

[3], Skoble et al., 2000

Supplemental Table 2. Median lethality of LADD and Lm-RIID strains.

**Strain**	**Description**	**LD_{50}**	**Reference**
WT Lm (DP-L4056)	Wild-type	1 x 10^5 CFU	Brockstedt et al., PNAS 2004; Lauer et al., 2002
LADD (Lm11)	Live attenuated	1 x 10^8 CFU	Brockstedt et al., PNAS 2004
BH3099	Live attenuated, Cre expression cassette	9.4 x 10^7 CFU	This study
LADD::pPL1-*actA* (BH5104)	Live attenuated, ActA complemented	1.5 x 10^5 CFU	This study
Lm-RIID (BH3226)	Lm-RIID, QuadVac cassette	2.2 x 10^8 CFU	This study
Lm-RIID::pPL1-*actA* (BH5108)	Lm-RIID, ActA complemented	1.2 x 10^8 CFU	This study
